# Evaluation of the diagnostic efficacy of ^18^F‐Fluorine‐2‐Deoxy‐D‐Glucose PET/CT for lung cancer and pulmonary tuberculosis in a Tuberculosis‐endemic Country

**DOI:** 10.1002/cam4.2770

**Published:** 2019-12-13

**Authors:** Alexandre Niyonkuru, Xiaomin Chen, Khamis Hassan Bakari, Dilani Neranjana Wimalarathne, Altine Bouhari, Maher Mohamad Rajab Arnous, Xiaoli Lan

**Affiliations:** ^1^ Department of Nuclear Medicine Union Hospital Tongji Medical College Huazhong University of Science and Technology Wuhan China; ^2^ Hubei Key Laboratory of Molecular Imaging Wuhan China

**Keywords:** ^18^F‐FDG PET/CT, diagnostic accuracy, lung cancer, pulmonary tuberculosis

## Abstract

**Objective:**

To determine the diagnostic efficacy of ^18^F‐FDG PET/CT in distinguishing between pulmonary tuberculosis (PTB) and lung cancer in solitary pulmonary nodule (SPN) in a country with a high prevalence of PTB.

**Methods:**

Patients with SPN who underwent ^18^F‐FDG PET/CT imaging were retrospectively included in the study. The final diagnosis was established by histopathology. A linear regression equation was fitted to a scatter plot of size and SUVmax of lung cancer and PTB. ROC was used to determine the optimal cutoff values and diagnostic accuracy of ^18^F‐FDG PET/CT in PTB and lung cancer.

**Results:**

About 514 patients were included with the mean age of 57.5 ± 10.6 years. Four hundred and seventy‐five cases were diagnosed as lung cancer, and 39 cases were PTB by histopathology. ^18^F‐FDG PET/CT had sensitivity, specificity, and diagnostic accuracy of 96.0%, 48.7%, and 92.0%, respectively. Utilization of SUVmax ≥2.5 in SPN resulted in 2 and 11 false positives cases of lung cancer and PTB, respectively, whereas SUVmax <2.5 resulted in 18 and 10 false‐positive cases of lung cancer and PTB, respectively. The SUVmax and the size of short‐axis in the lung cancer group were statistically higher than those in the PTB group. The linear regression equation parameters indicated the slope of the regression line of lung cancer was greater than that of PTB. The ROC curve demonstrated the SUVmax cutoff values of 4.85 and 2.25 for lung cancer and PTB, respectively for predicting the diagnostic accuracy of ^18^F‐FDG PET/CT.

**Conclusion:**

^18^F‐FDG PET/CT has a higher sensitivity and diagnostic accuracy for malignant SPN. However, it has high false‐positive rate and low specificity in tuberculosis endemic areas. Neither SUVmax nor the sizes of the nodules are valuable parameters for distinguishing between lung cancer and PTB. However, the SPN with larger short‐axis and higher SUVmax would be inclined to malignant tumor.

## Graphical abstract

18F‐FDG PET/CT has a higher sensitivity and diagnostic accuracy for malignant solitary pulmonary nodule. However, it has a high false positive rate and low specificity when diagnosing SPN in tuberculosis endemic areas.

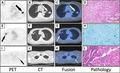

## 1 INTRODUCTION

Lung cancer is a leading cancer‐related death because of its high morbidity and mortality.^1^, ^2^, ^3^ Molecular/anatomic imaging with ^18^F‐fluorodeoxyglucose positron emission tomography/computed tomography (^18^F‐FDG‐PET/CT) has been well recognized as an important tool for detecting, identifying, and staging lung cancer. It provides metabolic information, which allows readers to distinguish between benign and malignant tissue. A maximum standardized uptake value (SUVmax) >2.5 on ^18^F‐FDG PET/CT has been widely accepted as a cutoff value for distinguishing between lung malignancies and benign diseases.^4^, ^5^ However, the specificity of ^18^F‐FDG PET/CT has been vigorously challenged. Some benign lesions, such as chronic infections, and infectious or inflammatory granulomatous lesions, especially tuberculosis,^6^, ^7^ sarcoidosis,^8^, ^9^, ^10^, ^11^ and inflammatory pseudotumor,^12^, ^13^ have been found to also be associated with increased uptake of ^18^F‐FDG, resulting in false‐positive lung cancer diagnoses. However, some types of cancer, such as carcinoid tumors^14^ and bronchoalveolar carcinoma (adenocarcinoma in situ)^15^ have low ^18^F‐FDG uptake, which can lead to false‐negative results.

Pulmonary Tuberculosis (PTB) is a common infectious disease‐causing serious medical and social problems.^16^ It is a chronic granulomatous infection caused by *Mycobacterium tuberculosis*; many developing countries have a high prevalence of PTB in their population due to weakening of the immune system.^17^ Globally, PTB is the second‐highest infectious cause of death after acquired immunodeficiency syndrome (AIDS).^18^ In 2015, tuberculosis (TB) was responsible for 9.6 million new cases of active PTB and 1.5 million deaths.^19^ The incidence rate of PTB per 100,000 population in Western countries such as UK, Iceland, and Denmark were 15, 3.5, and 7.4 per year, respectively.^20^ In China, the incidence rate of active PTB was 367 per 100,000 population in 2009, which ranks as the world's second largest number of cases, with 250,000 deaths annually.^21^

PTB is radiological one of the great mimickers of lung cancer,^22^, ^23^, ^24^ with a lot of clinical pictures and variants. In PTB‐endemic regions, it was observed that PTB resulted in 57.1%‐92.0% of false‐positive diagnoses of primary lung cancer and was also listed as one of the major false‐positive diagnoses of malignant lymph nodes.^25^, ^26^, ^27^, ^28^ The reason for these results is attributed to the nonspecificity of ^18^F‐FDG uptake. Inflammatory cells such as neutrophils, lymphocytes, and activated macrophages at the site of inflammation in PTB tend to have increased ^18^F‐FDG uptake and may be mistaken for foci of malignancy.^29^, ^30^, ^31^, ^32^, ^33^

Previous papers have reported on low specificity of ^18^F‐FDG PET/CT in geographical areas with a high prevalence of PTB. Li et al observed that PTB accounted for a high false‐positive rate (57.1% [8/14]) with ^18^F‐FDG PET alone.^25^ The sensitivity, specificity, diagnostic accuracy, and positive and negative predictive values for PTB with ^18^F‐FDG PET were 88.3%, 61.1%, 79.1%, 79.1%, and 75.9%, respectively, and on PET/CT were 96.7%, 75.0%, 88.5%, 88.1%, and 94.4%, respectively. Sathekge et al showed that the sensitivity and specificity of ^18^F‐FDG PET/CT for differentiating benign from malignant solitary pulmonary nodules (SPNs) in a TB‐endemic region were 85.7% and 25%, respectively.^26^

Some authors have indicated that neither the size of an SPN ≤3 cm nor the degree of uptake of ^18^F‐FDG with a SUVmax >2.5 can correctly distinguish PTB from malignancy. Khalaf et al in their study using an SUVmax cutoff of 2.5 observed that the sensitivity, specificity, and accuracy for diagnosing SPNs ≤1.0 cm in size were 85.0%, 36.0%, and 54.0%, respectively and for SPN size 1.1‐2.0 cm, they were 91.0%, 47.0%, and 79.0%, respectively. For SPNs 2.1‐3.0 cm they were 94.0%, 23.0%, and 76.0%, respectively.^34^

Other studies have shown that the SUVmax of PTB is higher compared with that of malignant lesions. Sathekge et al have shown that the mean SUVmax of PTB was 11.02 ± 6.6, while for malignant lesions the mean was 10.86 ± 8.9 *(P* = .0059*)*.^26^ Published reports have also highlighted that using an SUV cutoff of 2.5 in TB‐endemic regions results in low diagnostic accuracy compared with those using a suggested SUV cutoff of 5.0. du Toit et al concluded that the diagnostic accuracy of ^18^F‐FDG PET/CT in the evaluation of pulmonary lesions using an SUV cutoff point of 2.5 was very low, 71.4% compared to 86.7%, derived using an SUV cutoff of 5.0 in a TB‐endemic area.^35^

The high prevalence rate of PTB in this population may result in low diagnostic efficacy of ^18^F‐FDG PET/CT in the evaluation of SPNs. Up to now, there is no consensus about the use of ^18^F‐FDG PET/CT to distinguish pulmonary TB from lung malignancy**.** Due to the challenge of ^18^F‐FDG PET/CT imaging modality in differentiating malignant from benign SPN and consecutively to the controversial findings and results from the few studies conducted before on that matter of issue, we conducted this study by investigating a wide and detailed number of parameters to bring new information and comparing our findings to the previous ones.

We therefore hypothesized that is SUVmax or the size of the nodule helpful to the differential diagnosis of lung cancer and PTB? In this study, we retrospectively analyzed 514 patients who underwent ^18^F‐FDG PET/CT for the diagnosis of either lung cancer or PTB. Visual and semiquantitative analyses were used to assess PET/CT findings. Histopathology findings were considered as a gold standard for the final diagnosis. The main objective of this study was to evaluate the diagnostic efficacy of ^18^F‐FDG PET/CT imaging in SPN and to find out the clinical significance between lung cancer and PTB based on different parameters in China, a Tuberculosis‐endemic country.

## 2 PATIENTS AND METHODS

### 2.1 Patients

Permission to conduct this retrospective study was approved by the institution review board of Tongji Medical College, Huazhong University of Science and Technology.

About 519 cases of SPN who underwent ^18^F‐FDG PET/CT imaging between January 2013 and December 2016 were retrospectively evaluated. Inclusion criteria were as follows: (a) an SPN was defined as any solitary nodule appearing well‐circumscribed with a radiographic opacity diameter ≤3.0 cm in long axis and short axis and surrounded by aerated lung parenchyma; (b) histologically proven lung cancer or PTB. Exclusion criteria were: (a) presence of pulmonary disease other than lung cancer or PTB; (b) tumor metastases in the lung from other primary tumors in other sites of the body; (c) nodules >3.0 cm; (d) diabetes with blood glucose level >11 mmol/L.

Finally, 514 patients were enrolled in the study and five patients were excluded because they did not meet the inclusion criteria, including two SPN who had lung metastasis from other sites of the body, one SPN was sarcoidosis and two SPNs had sizes >3 cm. The cases included in the study had all undergone ^18^F‐FDG PET/CT imaging followed by biopsy or surgical removal of the SPN. Histological examination of the biopsy material or surgical specimens for pathology served as the gold standard for the diagnosis.

### 2.2 PET/CT acquisition

All patients fasted for at least 6 hours before the PET/CT examination. After ensuring a normal blood glucose level, patients received an intravenous injection of 0.10‐0.15 mCi/kg (3.7‐5.5 MBq/kg) of ^18^F‐FDG followed by resting for 50‐60 minutes before undergoing image acquisition. Imaging was performed using an integrated PET/CT (GE Discovery VCT, GE Healthcare, Milwaukee WI, USA). Low‐dose CT covering the area from the head to the pelvis was performed, and PET data were acquired in three‐dimensional mode; time per bed position was 2 minutes; 6‐8 bed positions were acquired.

After scatter and decay correction, PET data were reconstructed constantly with attenuation correction and redirected in axial, sagittal, and coronal slices. Coregistered images were displayed using Xeleris functional imaging software (GE), which enabled fused image analysis.

### 2.3 Image analysis: Visual assessment and semiquantitative analysis

Initially, images and the intensity of ^18^F‐FDG uptake by pulmonary lesions relative to the background activity in the uninvolved adjacent lung parenchyma and in the mediastinum were visually assessed.

To calculate SUVmax, manually defined regions of interest (ROIs) were drawn on the attenuation‐corrected emission images in the axial sections in which a suspicious lesion could be delineated. SUVmax values were generated for all the lesions included in this study.

PET, CT, and fused PET/CT images, as well as SUVmax, were independently reviewed and analyzed by two experienced nuclear medicine physicians and any disagreement was resolved by consensus. The semiquantitative analysis was based on the SUVmax and scored with a five‐point scale: 1 = definitely benign, 2 = probably benign, 3 = indeterminate, 4 = probably malignant, 5 = definitely malignant.^36^, ^37^

### 2.4 Statistical analysis

We employed commercial software (SPSS statistics version 20.0, SPSS, Inc, Chicago IL, USA) for statistical analysis. Categorical data were summarized in percentage; mean ± SD was used to describe continuous variables normally distributed while age range, SUVmax range, size range were used for continuous variables nonnormally distributed. Sensitivities, specificities, and diagnostic accuracies of ^18^F‐FDG PET/CT in SPN based on different parameters evaluated in the current study were also calculated. The Mann‐Whitney *U*‐test was used to compare the differences in continuous variables skewed distributed between lung cancer and PTB while Student's t‐test was used to compare continuous variables normally distributed. The Chi‐squared test was also used to compare the diagnostic value of different parameters of lung cancer and PTB in categorical variables while Fisher's exact test was used for categorical variables when a given parameter has <5 counts.

Receiver operating characteristics (ROC) analysis was used to compare the efficacy of ^18^F‐FDG PET/CT in predicting lung cancer and PTB. ROC curves were constructed to assess the area under the curve (AUC) and the optimal cutoff value of SUVmax for lung cancer and PTB. Linear regression analysis was used to evaluate the correlation between the SPN size (long‐axis diameter) and SUVmax in lung cancer and PTB. For all analyses, *P* < .05 was considered statistically significant. Pathology and PET/CT findings between lung cancer and PTB based on different parameters and their means ± SD were also compared using the same methods for categorical and continuous variables normally or nonnormally distributed.

## 3 RESULTS

A total of 514 cases (300 men [58.4%] and 214 women [41.6%]) with SPN were included and evaluated**.** The mean age was 57.5 ± 10.6 years old (range: 21‐84 years old). Thirty‐nine cases of PTB (7.6%) and 475 with lung cancer (92.4%) were finally diagnosed with histopathology. The lung cancers included adenocarcinoma (n = 377), squamous cell carcinoma (n = 26), adenosquamous carcinoma (n = 6), other non‐small cell lung cancer (n = 4), small cell lung cancer (n = 23), large cell lung cancer (n = 2), sarcoma (n = 4), carcinoid (n = 2), and unspecified lung cancer (n = 31).

### 3.1 Comparison of lung cancer and PTB based on pathology findings in different subgroups of cases

**Figure 1:**
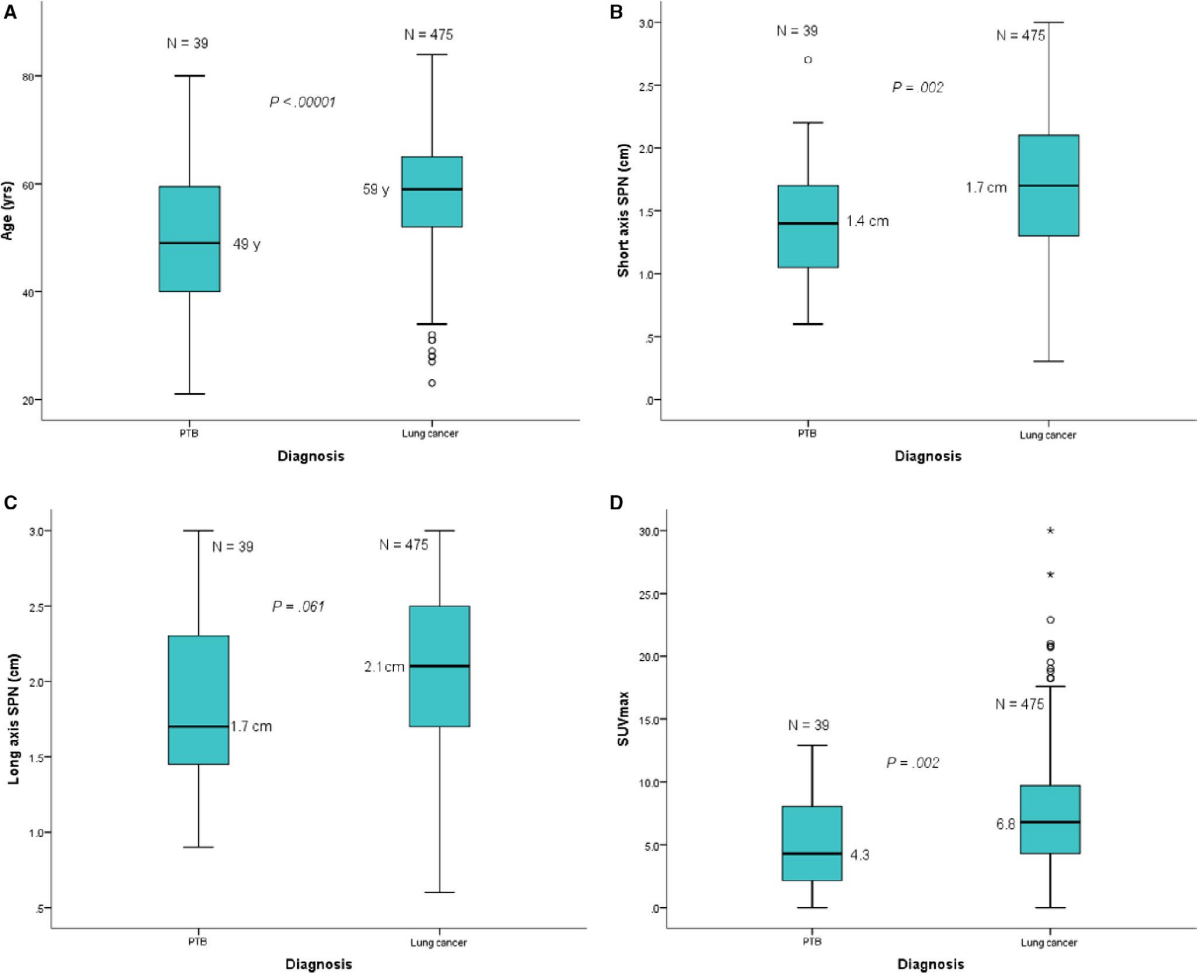
Box and whisker plots of lung cancer and pulmonary tuberculosis solitary pulmonary nodules distribution with their number, medians and *P* values, based on the age (A), short axis (B), long axis (C) and SUVmax (D). *P* < .05 was considered statistically significant. The distribution of the age (A), short axis (B), long axis (C) and SUVmax (D) for patients with PTB had a median and interquartile range (IQR: 25%‐75%) of 49 years (40‐60 years), 1.4 cm (1‐1.8 cm), 1.7 cm (1.4‐2.3 cm) and 4.3 (2.1‐8.2), respectively. For patients with lung cancer, the age, short axis, long axis, and SUVmax had a median and (IQR: 25%‐75%) as follows: 59 years (52‐65 years), 1.7 cm (1.3‐2.1 cm), 2.1cm (1.7‐2.5 cm), and 6.8 (4.3‐9.7), respectively

The basic characteristics of patients with PTB and lung cancer lesions and the comparison between lung cancer and PTB based on pathology findings are summarized in Table 1. Box and whisker plots of lung cancer and PTB data distribution for age, short‐axis, long‐axis, and SUVmax are depicted in Figure 1.

**Table 1:** Comparison between lung cancer and PTB based on pathology findings, n (%)

Parameters	Lung Cancer	PTB	*P*‐value
Sex			
Male	276 (58.11)	24 (61.54)	.6759
Female	199 (41.89)	15 (38.46)	
Age			
< 65years	341 (71.79)	37 (94.87)	.0017*
≥ 65years	134 (28.21)	2 (5.13)	
SUVmax			
< 2.5	48 (10.11)	12 (30.77)	.0001*
≥ 2.5	427 (89.89)	27 (69.23)	
Long Axis			
< 1cm	9 (1.89)	2 (5.13)	.1541
1‐1.9 cm	184 (38.73)	19 (48.72)	
2‐3 cm	282 (59.38)	18 (46.15)	
Short Axis			
<1 cm	31 (6.53)	5 (12.82)	.0045*
1‐1.9 cm	284 (59.79)	29 (74.36)	
2‐3 cm	160 (33.68)	5 (12.82)	

Abbreviation: SUVmax, Maximum Standardized Uptake value.

* *P* < .005 considered statistically significant.

The mean age of cases with pathologically proven malignant and benign SPN lesions was 58.2 ± 10.19 years (range 23‐84 years) and 49.1 ± 12.5 years (range 21‐80 years), respectively. The difference between the mean age of patients with lung cancer and patients with PTB was statistically significant (*P* < .0001). The 514 cases showed no statistically significant differences in sex or greatest diameter (long axis only) between lung cancer and PTB lesions (Table1). However, statistically significant differences could be seen in age (*P* = .0017), SUVmax (*P* = .0001) and short‐axis diameter (*P* = .0045) when comparing lung cancer and PTB.

The location of histological findings of lung cancer and PTB in different lobes of the lungs as well as of the features of PTB lesions are listed in the Table S1 and S2, respectively.

### 3.2 Comparison of mean of different parameters in pathologically proven lung cancer and PTB

Patients with lung cancer and PTB were divided by age, SUVmax, and nodule size subgroups. Information details and comparison of the mean ± SD between lung cancer and PTB are shown in Table 2. Statistically significant differences were found in the mean age of cases < 65 years (*P* = .001, *t* = 4.029) and in the total mean age of cases with lung cancer and PTB lesions (*P* = .0001, *t* = 5.278). In addition, a statistically significant difference was also observed in total mean size of the short axis of lung cancer and PTB lesions (1.69 ± 0.51 *vs* 1.43 ± 0.46; *P* = .002,* t* = 1.894). Overall, the SUVmax in the lung cancer group was statistically higher than that in the PTB group (7.42 ± 4.42 *vs* 5.27 ± 3.45; *P* = .003, *t* = 2.961), although not much difference exited in the subgroups.

**Table 2:** Comparison of the mean of parameters between Lung cancer and PTB pathologically proven

Parameters	Lung Cancer (n = 475)			PTB (n = 39)			*P*‐value	T‐value
	Number	Range	Mean ± SD	Number	Range	Mean ± SD		
Age (y)								
<65	341	23‐64	53.6 ± 7.8	37	21‐64	47.8 ± 11.1	.001*	4.029
≥65	134	65‐84	70 ± 4.1	2	65‐80	72.5 ± 10.6	.422	‐0.805
Total		23‐84	58.27 ± 10.20		21‐80	49.15 ± 12.26	.0001*	5.278
SUV max								
<2.5	48	0.0‐2.4	1.17 ± 0.8	12	0‐2.3	1.7 ± 0.6	.055	−1.96
≥2.5	427	2.5‐30	8.1 ± 4	27	3.3‐12.9	6.8 ± 2.9	.114	1.582
Total		0.0‐30	7.42 ± 4.42		0‐12.9	5.27 ± 3.45	.003*	2.961
Long Axis size (cm)								
<1	9	0.6‐0.9	0.8 ± 0.1	2	0.9‐0.9	0.9 ± 00	.33	−1.026
1‐1.9	184	1.0‐1.9	1.5 ± 0.2	19	1.1‐1.9	1.4 ± 0.2	.32	0.986
2‐3	282	2.0‐3.0	2.4 ± 0.3	18	2.0‐3.0	2.4 ± 0.3	.86	0.171
Total		0.6‐3.0	2.07 ± 0.56		0.9‐3.0	1.89 ± 0.60	.059	3.108
Short Axis size (cm)								
<1	31	0.3‐0.9	0.7 ± 0.1	5	0.6‐0.9	0.8 ± 0.1	.65	‐0.445
1‐1.9	284	1.0‐1.9	1.4 ± 0.2	29	1.0 −1.9	1.3 ± 0.2	.18	1.334
2‐3	160	2.0‐3.0	2.2 ± 0.2	5	2.1‐2.7	2.2 ± 0.2	.78	0.268
Total		0.3‐3.0	1.69 ± 0.51		0.60‐2.70	1.43 ± 0.46	.002*	1.894

Abbreviations: SUVmax, Maximum Standardized Uptake value; SD, Standard deviation.

* *P* < .005 considered statistically significant.

### 3.3 Visual analysis combined with semiquantitative analysis of ^18^F‐FDG PET/CT in the diagnosis of lung cancer

The visual and semiquantitative analysis revealed high sensitivity and accuracy and a low specificity of ^18^F‐FDG PET/CT in the diagnosis of lung cancer (Table 3). The overall sensitivity, specificity, and accuracy of ^18^F‐FDG PET/CT for the diagnosis of lung cancer were 96.0% (95% CI: 94%‐98%), 48.7% (95% CI: 33.0%‐64.4%), and 92.0% (95% CI: 89.6%‐94.3%), respectively. An SUVmax <2.5 had a sensitivity, specificity, and accuracy of 78.0%, 83.3%, and 78.0%, respectively, with two false‐positive lung cancers and 11 false‐positive PTB cases. An SUVmax ≥2.5 had a sensitivity, specificity, and accuracy of 97.0%, 33.3%, and 93.8%, respectively with 18 false‐positive lung cancers and 10 false‐positive PTB cases. The above findings indicate that SUVmax <2.5 has a high specificity in discriminating lung cancer from PTB while SUVmax ≥2.5 has a high sensitivity in differentiating lung cancer from PTB on ^18^F‐FDG PET/CT imaging.

**Table 3:** Evaluation of PET/CT in the diagnosis of Lung Cancer, Sensitivity, Specificity, and Accuracy

		PET/CT	Pathology [n, (%)]		Sensitivity% (95%CI)	Specificity% (95%CI)	Accuracy% (95%CI)
			PTB	Lung Cancer			
Overall Diagnosis	Benign	40	19 (48.7)	21 (4.4)	96.0 (94.0‐98.0)	48.7 (33.0‐64.4)	92.0 (89.6‐94.3)
	Malignant	474	20 (51.3)	454 (95.6)			
SUV max							
<2.5	Benign	21	10 (83.3)	11(22.9)	78.0 (67.0‐89.0)	83.3 (62.2‐100)	78.0 (67.9‐88.7)
	Malignant	39	2 (16.7)	37 (77.0)			
≥2.5	Benign	19	9 (33.3)	10 (2.3)	97.0 (96.0‐99.0)	33.3 (15.5‐51.1)	93.8 (91.6‐96.0)
	Malignant	435	18 (66.7)	417 (97.7)			
Long Axis‐Size							
<1cm	Benign	2	2 (100)	0 (0.0)	100 (100‐100)	100 (100‐100)	100 (100‐100)
	Malignant	9	0 (0.0)	9 (100)			
1‐1.9 cm	Benign	17	9 (47.3)	8 (4.3)	96.0 (93.0‐99.0)	47.3 (24.9‐69.8)	91.0 (87.2‐95.0)
	Malignant	186	10 (52.7)	176 (95.7)			
2‐3 cm	Benign	21	8 (44.4)	13 (4.6)	95.0 (93.0‐98.0)	44.4 (21.4‐67.4)	92.3 (89.3‐95.3)
	Malignant	279	10 (55.6)	269 (95.4)			
Short Axis‐Size							
<1cm	Benign	6	3 (60.0)	3 (9.7)	90.0 (81.0‐100)	60.0 (17.0‐100)	86.0 (74.8‐97.4)
	Malignant	30	2 (40.0)	28 (90.3)			
1‐1.9 cm	Benign	27	14 (48.3)	13 (4.6)	95.0 (93.0‐98.0)	48.2 (30.0‐66.4)	91.0 (87.8‐94.2)
	Malignant	286	15 (51.7)	271 (95.4)			
2‐3 cm	Benign	7	2 (40.0)	5 (3.1)	96.9 (94.0‐99.0)	40.0 (0.00‐82.9)	95.0 (91.8‐98.4)
	Malignant	158	3 (60.0)	155 (96.9)			

### 3.4 Linear regression analysis (correlation between SUVmax and size of nodules in lung cancer and PTB)

**Figure 2:**
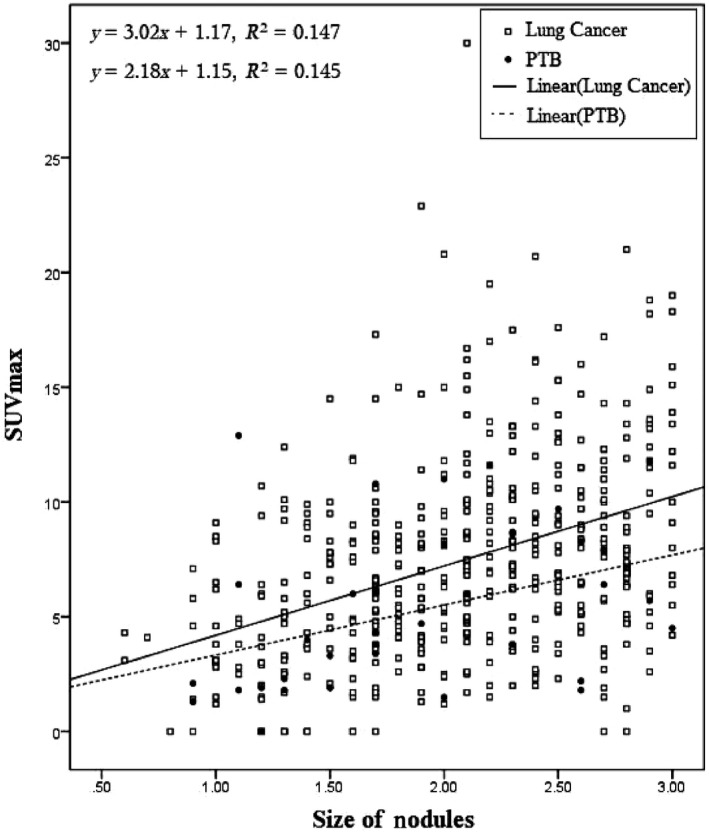
Linear regression of correlation equation between SUVmax and SPN size in lung cancer and PTB

A linear regression equation was generated to assess the correlation between the SUVmax and the size of nodules in lung cancer and PTB (Figure 2). For lung cancer SPN, the linear regression equation parameters and percentage of variance accounted for R^2^ were *y* = 3.0213 × +1.1735 and Adjusted‐R^2^ = 0.147, respectively. A statistically significant difference was observed between the SUVmax and the size of the nodules in lung cancer (*β* = 3.0213, 95% CI 2.36‐3.68, *P* < .0001). The linear regression equation parameters and R^2^ for PTB were *y* = 2.1795 × +1.153 and Adjusted‐R^2^ = 0.145, respectively. The difference between the SUVmax and the size of the nodules in PTB was statistically significant (*β* = 2.1795, 95% CI, 0.414‐3.945, *P* = .017). The equations and trendlines indicated that the slope of the regression line of lung cancer was greater than the regression line of PTB. The distribution of nodules in the plot shows that lung cancer nodules with larger size had a higher probability of having a higher SUVmax. In addition, for PTB, the nodules are randomly distributed throughout the plot. The percentage of variance in both lung cancer (Adjusted‐R^2^ = 0.147) and PTB (Adjusted‐R^2^ = 0.145) were found to be almost the same, indicating difficulty in discriminating lung cancer from PTB.

**Figure 3:**
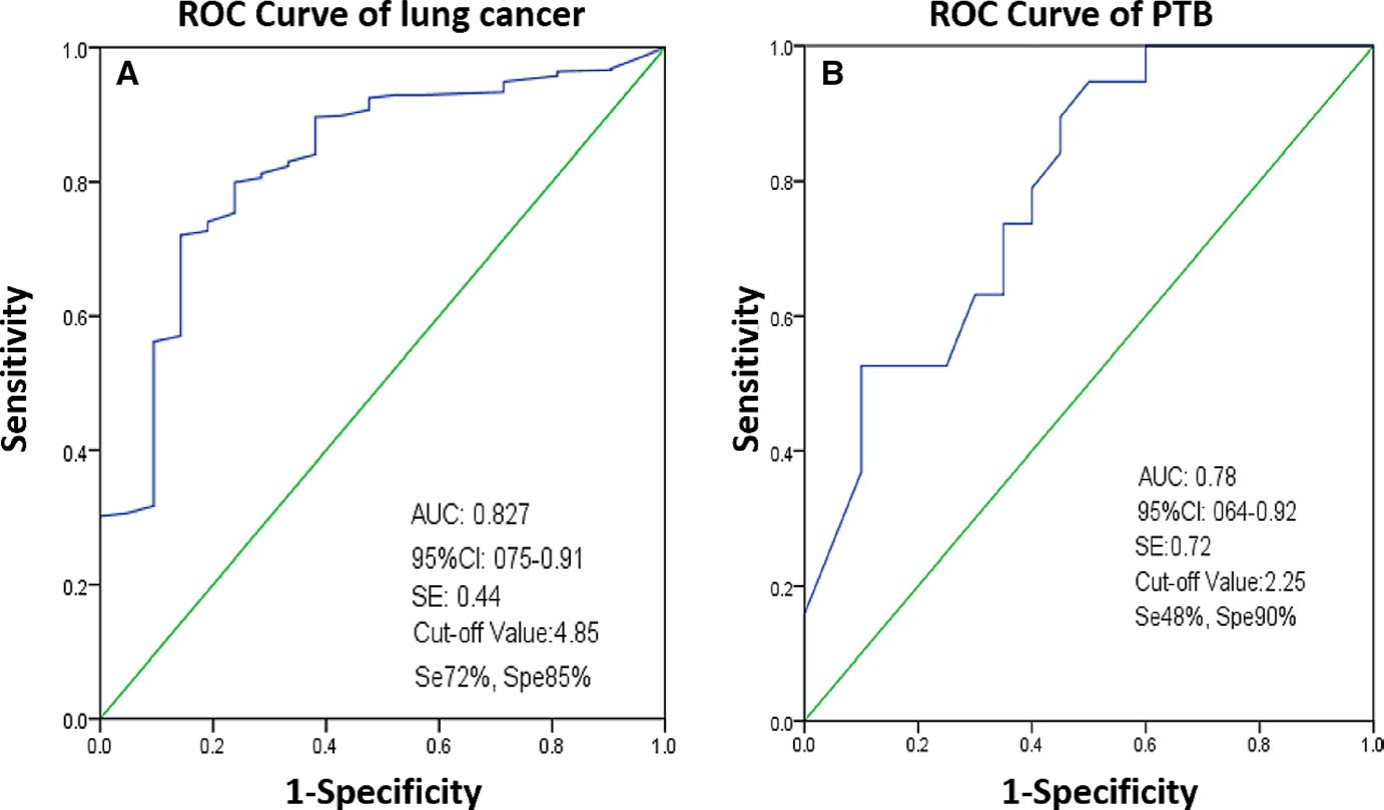
Receiver operating curves of ^18^F‐FDG PET/CT in predicting lung cancer (A) and pulmonary tuberculosis (B) based on SUVmax values (Abbreviations: AUC, Area under the curve; Se, sensitivity; Spe, specificity; SE, Standard Error).

From the ROC curves (Figure 3), the cutoff value for positive ^18^F‐FDG PET/CT lung cancer was 4.85 with sensitivity, specificity, and AUC of 72.0%, 85.0%, and 0.827 (95% CI: 0.75‐0.91), respectively. The ROC curve of ^18^F‐FDG PET/CT for predicting PTB had a cutoff of 2.25 with sensitivity, specificity, and AUC of 48.0%, 90.0% and 0.78 (95% CI: 0.64‐0.92), respectively. The AUC for lung cancer was greater than that of PTB, 0.827 vs 0.784, respectively.

**Figure 4:**
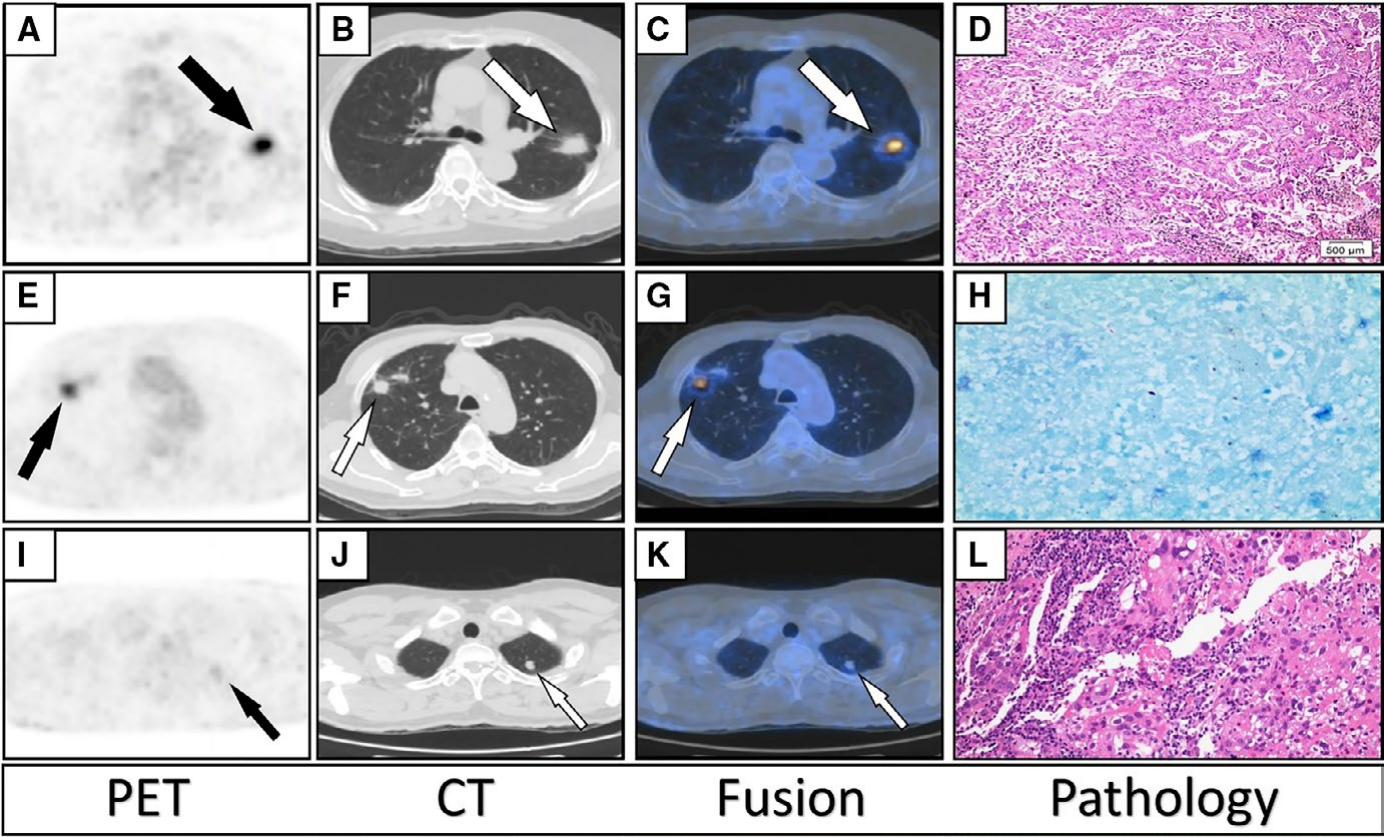
A–D, axial PET, CT, fused PET/CT, and pathology images of a 68 year‐old‐female patient diagnosed with adenocarcinoma on ^18^F‐FDG PET/CT in the upper lobe of the left lung, confirmed by pathology analysis resulting in true‐positive lung cancer; SUVmax:17.5, SA: 2 cm and LA: 2.3 cm. E–H, axial PET, CT, fused PET/CT and pathology images of an 8‐ year‐old male patient diagnosed with lung cancer on ^18^F‐FDG PET/CT in the upper lobe of the right lung but showing PTB on pathology analysis (false‐positive lung cancer); SUVmax: 6, SA: 1.6 cm, LA: 2.1 cm. I–L, axial PET, CT, fused and pathology images of 48‐year‐old male patient diagnosed with PTB on ^18^F‐FDG PET/CT in the upper lobe of the left lung with pathology findings of adenocarcinoma of the lung (false‐negative lung cancer); SUVmax: 2, SA: 0.8 cm, LA: 1.2 cm. (Abbreviations: PET/CT, Positron Emission Tomography/Computed Tomography; FDG, Fluorine‐2‐Deoxy‐ D‐glucose; PTB, Pulmonary tuberculosis; SUVmax, maximum Standardized Uptake value; SA, Short Axis; LA, Long axis)

**Figure 5:**
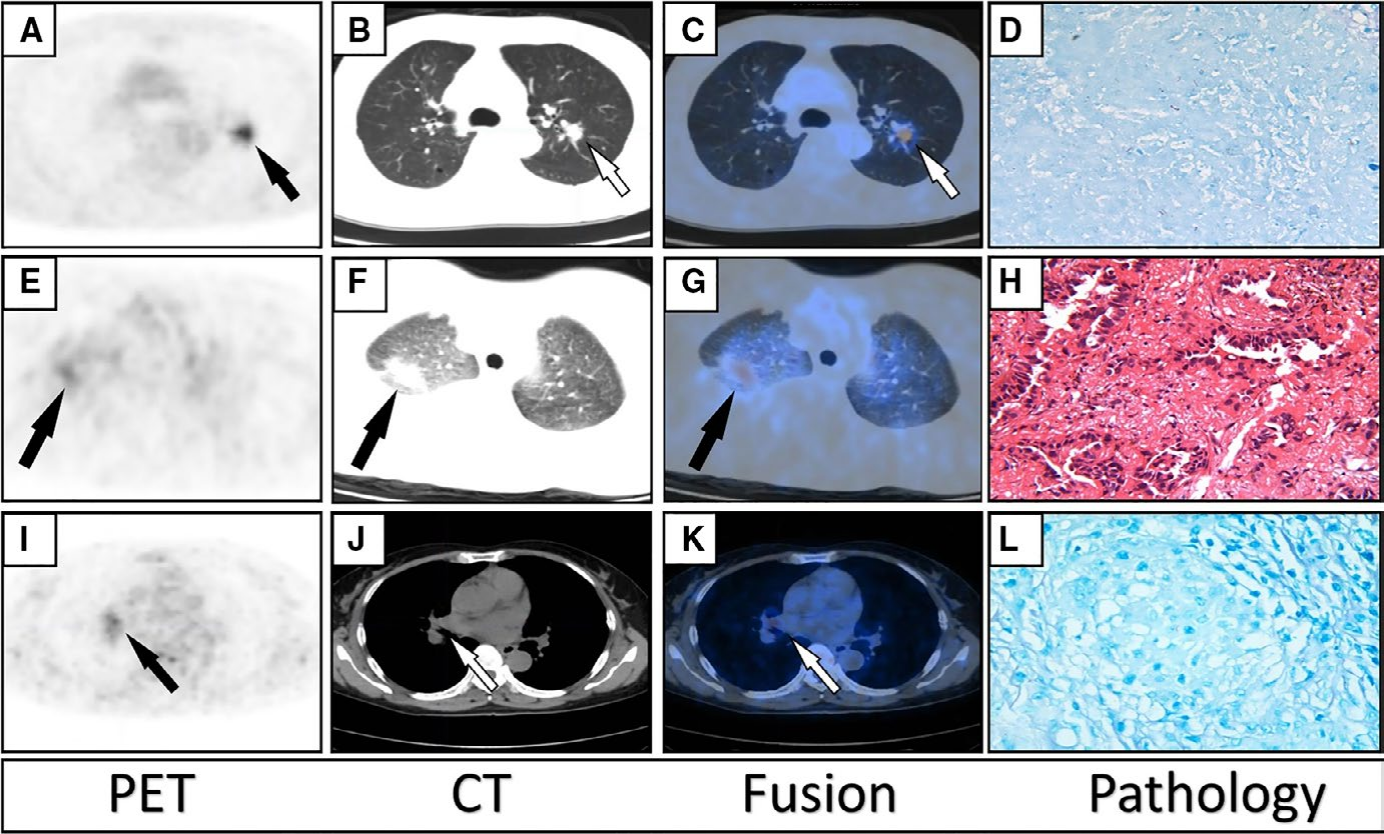
A–D, axial PET, CT, fused PET/CT images, and pathology of a 53 year‐old‐male patient diagnosed with PTB on ^18^F‐FDG PET/CT in the upper lobe of the left lung and confirmed by pathology analysis as PTB (true‐positive PTB); SUVmax: 6.1, SA: 1.5 cm, LA: 1.7 cm. E–H, axial PET, CT, fused PET/CT and pathology images of a 44‐year‐old female patient diagnosed as PTB on ^18^F‐FDG PET/CT in the middle lobe of the right lung with pathologic diagnosis of an adenocarcinoma, resulting in false‐positive PTB; SUVmax: 3.6, SA: 1.7cm, LA: 2.7 cm. I–L, axial PET, CT, fused PET/CT, and pathology images of a 51‐year‐old female patient diagnosed as lung cancer on ^18^F‐FDG PET/CT in the lower lobe of the right lung with a pathology diagnosis of PTB ( false‐negative PTB); SUVmax: 4, SA: 1.2 cm, LA: 1.4 cm**.** (Abbreviations: PET/CT, Positron Emission Tomography/Computed Tomography; FDG, Fluorine‐2‐Deoxy‐ D‐glucose; PTB, Pulmonary tuberculosis; SUVmax, Maximum Standardized Uptake value; SA, Short Axis, LA, Long axis)

Figure 4 and Figure 5 demonstrated three different cases with true‐positive, false‐positive, and false‐negative lung cancer and PTB images on ^18^F‐FDG PET/CT, respectively.

## 4 DISCUSSION

In this retrospective study, we found that ^18^F‐FDG PET/CT has a higher sensitivity and diagnostic accuracy for malignant SPN. However, it has some difficulty in differential diagnosis with PTB in tuberculosis endemic areas because of high false‐positive rate. Although neither SUVmax nor the sizes of the nodules are valuable parameters for distinguishing between lung cancer and PTB, the SPNs with larger short‐axis and higher SUVmax would be inclined to malignant tumors. Moreover, PET/CT diagnosis of small nodules <1 cm tended to have higher specificity than that for larger nodules for both short‐axis and long‐axis diameter on ^18^F‐FDG PET/CT imaging for the diagnosis of lung cancer, while nodules with greater size tended to show a higher sensitivity and diagnostic accuracy.

The biggest challenge for ^18^F‐FDG PET/CT modality imaging is in differentiating benign from malignant solitary pulmonary nodules (SPN). Combination of metabolic (PET component) and anatomic (CT component) imaging is synergistic by maintaining sensitivity of CT and specificity of PET, resulting in an overall significantly improved accuracy with 97% sensitivity and 85% specificity, for differentiating malignant from benign pulmonary nodules,^37^, ^38^ but this modality imaging is critical to deal with the false‐positive and false‐negative ^18^F‐FDG PET/CT imaging in SPN. Pulmonary tuberculosis by mycobacterium has been reported to accumulate ^18^F‐FDG. Invasion of the pulmonary alveoli with mycobacteria signals the start of TB infection which later on invades and replicates within the alveolar macrophages.^39^ Inflammatory cells such as neutrophils and activated macrophages at the site of inflammation tend to have more ^18^F‐FDG uptake.^29^, ^30^, ^31^, ^32^, ^33^ Earlier studies have indicated that macrophages and lymphocytes in TB granulomas are responsible for high ^18^F‐FDG uptake on PET imaging.^29^, ^30^, ^31^, ^32^, ^33^

Our study revealed overall sensitivity, specificity, and diagnostic accuracy of 96.0%, 48.7%, and 92.02%, respectively of ^18^F‐FDG PET/CT in diagnosing lung cancer. These findings are similar to previous findings by Martins et al^35^ and Kim et al^34^ who described their sensitivity and diagnostic accuracy to be 92.9%‐97% and 81.2%‐93%, respectively, but with a higher specificity of 72.2% and 85.0%, respectively. The difference can be attributed to the fact that their studies were conducted in a nonendemic PTB region.

The sensitivity, specificity, and diagnostic accuracy of ^18^F‐FDG PET/CT for SUVmax ≥2.5 were 97.0%, 33.3%, and 93.8%, while for SUVmax <2.5 were 78.0%, 83.3% and 78.0%, respectively. These findings indicate that SUVmax ≥2.5 had higher sensitivity and diagnostic accuracy than SUVmax <2.5 [(97.0% vs 78.0%, *P* < .0001) and (93.8% vs 78.0%, *P* < .0001), respectively] in the diagnosis of lung cancer. In contrast, SUVmax <2.5 showed a higher specificity than SUVmax ≥2.5 (83.3% vs 33.3, *P* < .0001), respectively. Findings similar to our results were expressed by Chen et al^36^ and Huang et al^37^ who observed that the sensitivity, specificity, and diagnostic accuracy of SUVmax ≥2.5 in their studies were 83%/97%, 48%/38%, and 72%/82%, respectively.

Semiquantitatively analysis of ^18^F‐FDG PET/CT scan by using a maximum standardized uptake value (SUVmax), which is a reflection degree of ^18^F‐FDG uptake and has widely been used to differentiate benign lung lesions from malignancies. The SUV is the ratio of activity in tissue per unit volume to the activity of the injected dose per patient body weight. Bryant et al found that the maximum SUV is a predictor of pathology. The higher the SUVmax, the higher the chance a nodule would be malignant.^40^ There is variation in threshold SUVmax used among different institutions for differentiating benign from malignant lesions. An SUVmax of 2.5 appeared to be a relatively good cutoff for the diagnosis of lung cancer^41^; however, being affecting by a larger number of parameters (such as used equipment, the physics, biological factors), which are difficult to control and specify, the SUVmax semiquantitative analysis cannot be used as reliable parameter in differentiating lung cancer from PTB nodules.

In this study, when an SUVmax ≥2.5 was used to classify SPNs, two and 11 false‐positive lung cancer and PTB diagnoses resulted, respectively. Also, utilization of SUVmax <2.5 in SPNs resulted in 18 false‐positive lung cancer and 10 false‐positive PTB diagnoses. The data shows that SUVmax values are not a valuable tool in assessing solitary pulmonary nodules in PTB‐endemic areas.

When considering the long‐axis diameter of an SPN in distinguishing lung cancer from PTB, the diagnostic efficacy of ^18^F‐FDG PET/CT was revealed to have decreasing specificity as with increasing long‐axis diameter 100% for group 1 (< 1 cm), 47.4% for group 2 (1‐1.9 cm), and 44.4% for group 3 (2‐3 cm). The high sensitivity, specificity, and diagnostic accuracy observed in group 1 (<1 cm) were probably due to the smaller number of nodules in this group (11 out of 514, [2.1%]). The same findings in the specificity of ^18^F‐FDG PET/CT were observed for the short‐axis diameter of nodules. With respect to the size of the nodules, numerous SPNs assessed by both short and long‐axis diameter presented with 20 and 21 false‐positive cases of lung cancer and PTB, respectively. Regarding sensitivity and diagnostic accuracy, these findings are congruent with previous report by Khalaf et al 31 which describes that their sensitivity and diagnostic accuracy increased with increasing size of the nodule from 85.0% (<1.0 cm) to 94.0% (2.1‐3.0 cm) and from 54% (< 1.0 cm) to 76.0% (2.1‐3.0 cm), respectively. Conversely, their specificity decreased with increasing size, declining from 47.0% (1.1‐2.0 cm) to 23% (2.1‐3.0 cm).

From the findings in this study, although significant differences in short‐axis size of the nodules between PTB and lung cancer patients were observed (*P* = .002), the diagnosis of lung cancer and PTB was not significantly influenced by the size of the nodules. The difference in the size of SPNs ≤3 cm could not be used as a criterion for distinguishing between lung cancer and PTB. Small lesions (˂1 cm) are challenging due to the limited spatial resolution of PET, which is approximately 5‐7 mm for modern scanners and no specific uptake of FDG in small SPN.

The linear regression equation and R^2^ for lung cancer and PTB as well as the trendlines for both diseases showed that the slope of the regression line was greater for lung cancer than for PTB. For lung cancer, a statistically significant difference was observed between the SUVmax and the size of the nodules (*β* = 3.0213, 95% CI: 2.36‐3.68, *P* < .0001), indicating that an increase of 1cm in nodule size results in a threefold increase in SUVmax. For PTB difference between the SUVmax and size of the nodules was statistically significant (*β* = 2.1795, 95% CI: 0.414‐3.945, *P* = .017), showing that an increase of 1cm in nodule size results to approximately twofold increase in SUVmax. From Figure 2, it can be seen that from the left side where the nodules are small (<1 cm) through the middle to the right side where nodules are larger (≥2 cm), all the nodules for lung cancer and PTB are plotted and mixed randomly with a predominance of lung cancer nodules in the middle and right‐sided areas. From the plot, there was no SUVmax value cutoff to separate lung cancer nodules from PTB nodules, but nodules for both diseases were plotted and polarized in the middle portion of the plot where SUVmax is ≥2.5 and the size >1.50 cm. Fewer PTB nodules appear in the upper portion of the plot where the SUVmax >10, particularly for larger nodules ≥2 cm. Neither the SUV max nor the size of the nodule can be used to distinguish between lung cancer and PTB in the case of SPN.

Based on the ROC curve, the AUC and SUVmax cutoff of ^18^F‐FDG‐PET/CT for lung cancer was greater than those of PTB, 0.827 vs 0.784, and 4.85 vs 2.25, respectively. Our findings indicated that the AUC for lung cancer, 0.827 (95% CI 0.74‐0.91) was similar to that described in a previous study by Martins et al^35^ In another study, the AUC to determine the best SUVmax cutoff for diagnosis of lung cancer was 0.877 (95% CI 0.75‐–0.99).^36^ Their values are slightly higher compared with our findings due to the fact that our study was conducted in a PTB‐endemic area.

The study has limitations. First, data were collected from a single center. The second limitation is the selection bias since this was a retrospective study, which enlisted only patients with pathologically proven SPN. The third limitation is the lack of other granulomatous diseases in our study. Furthermore, CT scan features of SPNs, including centrilobular nodules and branching linear structures (tree in‐bud‐appearance), well‐defined segmental or lobular consolidation with enlargement of lymph nodes in the hilum or the mediastinum, internal cavitation, and bronchial wall thickening in active post‐primary PTB,^42^ and features such as location, shape, calcification, vacuolation, pleural indentation, borders, surrounding ground‐glass opacification (sGGO), satellite opacification, vessel convergence, enlarged lymph nodes, or masses in other organs that might suggest primary or secondary lung cancer,^43^, ^44^ may be complementary for the differentiation of PTB and lung cancer on ^18^F‐FDG‐PET/CT imaging. Future multicenter prospective studies with larger proportions of active tuberculosis and granulomatous nodules should be conducted to validate our findings. Other PET radiotracers such as ^11^C‐choline, ^18^F‐FEC, ^18^F‐FLT, ^68^Ga‐Citrate that are specific for lung cancer and PTB, and which may increase the sensitivity and specificity of differentiating SPNs, should also be considered.

## 5 CONCLUSION

Although ^18^F‐FDG PET/CT has a higher sensitivity and diagnostic accuracy for malignant SPN, it is still challenging because of high false‐positive rate in PTB‐endemic regions. Neither SUVmax nor the sizes of the nodules are valuable parameters for distinguishing between lung cancer and PTB. However, the SPNs with larger short‐axis and higher SUVmax would be inclined to malignant tumors. Future multicentered prospective studies with larger proportions of active tuberculosis and granulomatous nodules should be conducted to validate our findings.

## CONFLICT OF INTEREST

The authors declare that there was no conflict of interest.

## IRB STATEMENT

This retrospective study of existing patient data and images was approved by the institutional review board of Union Hospital, Tongji Medical College, Huazhong University of Science and Technology. The requirement for informed consent was waived.

## Data Availability

I confirm that my article contains a Data Availability Statement even if no data is available (list of sample statements) unless my article type does not require one. I confirm that I have included a citation for available data in my references section, unless my article type is exempt.
