# Supplementary material for: Evaluation of the diagnostic efficacy of 18F‐Fluorine‐2‐Deoxy‐D‐Glucose PET/CT for lung cancer and pulmonary tuberculosis in a Tuberculosis‐endemic Country
Source: Cancer Med. 2019 Dec 13;9(3):931–42. doi: 10.1002/cam4.2770 (PMC6997090; doi:10.1002/cam4.2770)
Supplement: Supplementary file 1 [file CAM4-9-931-s001.docx]

**Supplementary Data**

**Title:** Evaluation of the diagnostic efficacy of ^18^F-Fluorine-2-Deoxy-D-Glucose PET/CT for Lung Cancer and Pulmonary Tuberculosis in tuberculosis-endemic country.

**Authors:** Alexandre Niyonkuru M.S ^1,2#^, Xiaomin Chen M.S ^1,2#^, Khamis Hassan Bakari M.S ^1,2^, Dilani Neranjana Wimalarathne M.S ^1,2^, Altine Bouhari M.S ^1,2^, Maher Mohamad Rajab Arnous M.S ^1,2^ , Xiaoli Lan, MD, PhD ^1,2*^

^1^ Department of Nuclear Medicine, Union Hospital, Tongji Medical College, Huazhong University of Science and Technology, Wuhan, Hubei Province 430022;

^2^ Hubei Key Laboratory of Molecular Imaging, Wuhan, Hubei Province 430022, China

# Alexandre Niyonkuru and Xiaomin Chen contributed equally to the manuscript.

^*^ Corresponding author: Xiaoli Lan, M.D, Ph.D

**Address:** Department of Nuclear Medicine, Union Hospital, Tongji Medical College, Huazhong University of Science and Technology, No. 1277 Jiefang Ave, Wuhan, Hubei Province 430022, China.

E-mail address: [LXL730724@hotmail.com](mailto:LXL730724@hotmail.com)

**Table S1** The location of histological findings of lung cancer and PTB in different lobes of the lungs

|  | Right lung | | | Left lung | | Total |
| --- | --- | --- | --- | --- | --- | --- |
| **Histological type** | Upper lobe | Middle lobe | Lower lobe | Upper lobe | Lower lobe |  |
| Adenocarcinomas | 125 | 32 | 63 | 94 | 59 | 373* |
| Squamous cell carcinomas | 8 | 1 | 9 | 5 | 3 | 26 |
| Adenosquamous cell carcinomas | 1 | 1 | 1 | 1 | 2 | 6 |
| Other NSCLC | 1 | - | 1 | 2 | - | 4 |
| SCLC | 4 | 2 | 4 | 9 | 3 | 22* |
| LCLC | 1 | - | - | 1 | - | 2 |
| Sarcoma | 1 | - | 1 | 1 | 1 | 4 |
| Carcinoid tumor | - | - | 1 | 1 | - | 2 |
| Other lung cancers | 8 | 1 | 7 | 9 | 6 | 31 |
| Tuberculosis | 13 | 3 | 3 | 9 | 11 | 39 |

(*Four adenocarcinomas were not mentioned for their lung and lobe locations and *One SCLC was not mentioned for its lung and lobe location

*Abbreviations: NSCLC= Non small cell lung cancer; SCLC= Small cell lung cancer, LCLC= Large cell lung cancer.*

**Table S2** PTB lesions characteristics

| **No** | Sex | | Age | SA SPN size | LA SPN size | SUV Max | PET/CT dc | Pathology dc | SPN calcification | Axil.L N assoc | Mediastinal Nodes |
| --- | --- | --- | --- | --- | --- | --- | --- | --- | --- | --- | --- |
| **1** | | 1 | 55 | 1.8 | 2.6 | 2.2 | B | B | No | No | Yes |
| **2** | | 2 | 40 | 1.6 | 2.3 | 3.8 | B | B | No | Yes | No |
| **3** | | 1 | 36 | 2.1 | 3 | 4.5 | B | B | No | No | No |
| **4** | | 2 | 48 | 1.3 | 1.5 | 1.9 | B | B | Yes | No | No |
| **5** | | 2 | 63 | 1.5 | 2 | 1.5 | B | B | No | No | No |
| **6** | | 1 | 49 | 1.2 | 1.7 | 3.4 | M | B | No | Yes | Yes |
| **7** | | 1 | 44 | 1.2 | 1.5 | 3.3 | M | B | Yes | Yes | Yes |
| **8** | | 1 | 59 | 2.1 | 2.3 | 3.8 | M | B | No | Yes | Yes |
| **9** | | 2 | 60 | 1.4 | 1.7 | 10.8 | M | B | No | No | Yes |
| **10** | | 1 | 30 | 1 | 1.1 | 12.9 | M | B | No | No | No |
| **11** | | 1 | 53 | 0.9 | 1.3 | 1.8 | M | B | No | Yes | Yes |
| **12** | | 1 | 60 | 1.6 | 2.3 | 8.7 | M | B | Yes | No | Yes |
| **13** | | 1 | 46 | 1.8 | 2.2 | 11.6 | M | B | No | No | No |
| **14** | | 2 | 40 | 1.3 | 1.5 | 1.9 | B | B | No | Yes | No |
| **15** | | 2 | 48 | 1.2 | 1.7 | 3.4 | M | B | No | Yes | Yes |
| **16** | | 1 | 64 | 2.2 | 2.6 | 1.8 | B | B | Yes | Yes | Yes |
| **17** | | 2 | 51 | 1.2 | 1.4 | 4 | M | B | No | Yes | Yes |
| **18** | | 2 | 44 | 0.8 | 1.6 | 6 | B | B | Yes | Yes | No |
| **19** | | 1 | 40 | 1.1 | 1.2 | 0 | B | B | Yes | No | No |
| **20** | | 1 | 21 | 1 | 1.1 | 1.8 | B | B | Yes | No | Yes |
| **21** | | 1 | 62 | 1.9 | 1.9 | 4.7 | M | B | Yes | No | Yes |
| **22** | | 2 | 56 | 1 | 1.1 | 6.4 | M | B | No | No | Yes |
| **23** | | 1 | 33 | 1.8 | 2.5 | 9.7 | M | B | No | No | Yes |
| **24** | | 1 | 53 | 1.5 | 1.7 | 6.1 | B | B | No | No | Yes |
| **25** | | 1 | 37 | 1.1 | 2.7 | 6.4 | B | B | No | Yes | No |
| **26** | | 1 | 46 | 0.6 | 0.9 | 1.3 | B | B | Yes | Yes | Yes |
| **27** | | 1 | 80 | 1.6 | 2.1 | 6 | M | B | No | Yes | Yes |
| **28** | | 2 | 53 | 0.9 | 1.2 | 1.9 | M | B | No | No | Yes |
| **29** | | 1 | 32 | 1.6 | 2 | 8.2 | M | B | No | No | Yes |
| **30** | | 2 | 60 | 1 | 1.3 | 2.3 | B | B | No | Yes | Yes |
| **31** | | 2 | 61 | 1 | 1.7 | 3.8 | B | B | Yes | No | Yes |
| **32** | | 2 | 47 | 1.6 | 2.3 | 8.6 | M | B | Yes | No | No |
| **33** | | 1 | 37 | 0.8 | 0.9 | 2.1 | B | B | No | Yes | No |
| **34** | | 1 | 65 | 1.5 | 2.9 | 5.7 | B | B | Yes | Yes | Yes |
| **35** | | 2 | 61 | 1.4 | 2 | 11 | M | B | No | Yes | Yes |
| **36** | | 1 | 48 | 2.1 | 2.7 | 7.9 | M | B | No | No | Yes |
| **37** | | 2 | 27 | 1.8 | 2.6 | 8.3 | B | B | No | Yes | Yes |
| **38** | | 1 | 51 | 1.5 | 1.7 | 4.3 | B | B | Yes | No | Yes |
| **39** | | 1 | 57 | 2.7 | 2.9 | 11.8 | M | B | Yes | Yes | Yes |

Abbreviations: Sex (1=male, 2= female), M= malignant, B=Benign; SA= short Axis, LA= long axis, SPN= Solitary pulmonary nodules; SUVmax = Maximun Standardized uptake value; Axil L.N Assoc. = axillary lymph nodes associated.
